# Data Management and Privacy Policy of COVID-19 Contact-Tracing Apps: Systematic Review and Content Analysis

**DOI:** 10.2196/35195

**Published:** 2022-07-12

**Authors:** Marco Bardus, Melodie Al Daccache, Noel Maalouf, Rayan Al Sarih, Imad H Elhajj

**Affiliations:** 1 Institute of Applied Health Research College of Medical and Dental Sciences University of Birmingham Birmingham United Kingdom; 2 Department of Health Promotion and Community Health Faculty of Health Sciences American University of Beirut Beirut Lebanon; 3 Center for Research on Population and Health Faculty of Health Sciences American University of Beirut Beirut Lebanon; 4 Department of Electrical and Computer Engineering School of Engineering Lebanese American University Byblos Lebanon; 5 Department of Electrical and Computer Engineering Maroun Semaan Faculty of Engineering and Architecture American University of Beirut Beirut Lebanon

**Keywords:** COVID-19, mobile applications, contact tracing

## Abstract

**Background:**

COVID-19 digital contact-tracing apps were created to assist public health authorities in curbing the pandemic. These apps require users’ permission to access specific functions on their mobile phones, such as geolocation, Bluetooth or Wi-Fi connections, or personal data, to work correctly. As these functions have privacy repercussions, it is essential to establish how contact-tracing apps respect users’ privacy.

**Objective:**

This study aimed to systematically map existing contact-tracing apps and evaluate the permissions required and their privacy policies. Specifically, we evaluated the type of permissions, the privacy policies’ readability, and the information included in them.

**Methods:**

We used custom Google searches and existing lists of contact-tracing apps to identify potentially eligible apps between May 2020 and November 2021. We included contact-tracing or exposure notification apps with a Google Play webpage from which we extracted app characteristics (eg, sponsor, number of installs, and ratings). We used Exodus Privacy to systematically extract the number of permissions and classify them as *dangerous* or *normal*. We computed a Permission Accumulated Risk Score representing the threat level to the user’s privacy. We assessed the privacy policies’ readability and evaluated their content using a 13-item checklist, which generated a Privacy Transparency Index. We explored the relationships between app characteristics, Permission Accumulated Risk Score, and Privacy Transparency Index using correlations, chi-square tests, or ANOVAs.

**Results:**

We identified 180 contact-tracing apps across 152 countries, states, or territories. We included 85.6% (154/180) of apps with a working Google Play page, most of which (132/154, 85.7%) had a privacy policy document. Most apps were developed by governments (116/154, 75.3%) and totaled 264.5 million installs. The average rating on Google Play was 3.5 (SD 0.7). Across the 154 apps, we identified 94 unique permissions, 18% (17/94) of which were dangerous, and 30 trackers. The average Permission Accumulated Risk Score was 22.7 (SD 17.7; range 4-74, median 16) and the average Privacy Transparency Index was 55.8 (SD 21.7; range 5-95, median 55). Overall, the privacy documents were difficult to read (median grade level 12, range 7-23); 67% (88/132) of these mentioned that the apps collected personal identifiers. The Permission Accumulated Risk Score was negatively associated with the average App Store ratings (*r*=−0.20; *P*=.03; 120/154, 77.9%) and Privacy Transparency Index (*r*=−0.25; *P*<.001; 132/154, 85.7%), suggesting that the higher the risk to one’s data, the lower the apps’ ratings and transparency index.

**Conclusions:**

Many contact-tracing apps were developed covering most of the planet but with a relatively low number of installs. Privacy-preserving apps scored high in transparency and App Store ratings, suggesting that some users appreciate these apps. Nevertheless, privacy policy documents were difficult to read for an average audience. Therefore, we recommend following privacy-preserving and transparency principles to improve contact-tracing uptake while making privacy documents more readable for a wider public.

## Introduction

### Strategies to Contain COVID-19

Since the beginning of the COVID-19 pandemic in early 2020, in the absence of vaccines or pharmacological treatments for the SARS-CoV-2 virus, some researchers have urged governments and the global public health community to speed up the response to contain SARS-CoV-2, pushing for the implementation of integrated nonpharmaceutical or nonpharmacological interventions (NPIs) [1]. Traditionally, NPIs adopted to curb an epidemic or pandemic such as influenza include mandating personal protective measures among health care professionals and citizens (eg, wearing masks), environmental measures such as isolating or quarantining positive cases [2], physical distancing, lockdowns, and travel restrictions [3]. However, more than two years into the pandemic, even after several vaccines were developed and rolled out worldwide, many countries have struggled to effectively and efficiently implement NPIs. In the absence of aggressive testing, contact tracing, and quarantining, an early study suggested that the only way to control COVID-19 would have included intermittent lockdowns until *herd immunity* was built up, resulting in unnecessary deaths [4]. Unfortunately, this seems to have been the case in many countries of the world, which observed alarming surges in cases of the SARS-CoV-2 virus. As of April 2021, the virus had infected >130 million individuals and claimed the lives of >2.8 million worldwide [5]. NPIs require significant investments in human resources and equipment and a level of coordination that might not be feasible in all contexts. For example, many low- and middle-income countries do not have the resources to enforce containment and testing policies [6] or purchase large amounts of vaccines. In addition, quarantining and physical distancing may not work among underprivileged and vulnerable segments of the population relying on daily wages for survival [7].

Contact tracing is one of the most cost-efficient NPIs available to break the chain of viral transmission [8]. According to the interim guidance of the World Health Organization, contact tracing consists of systematically identifying individuals exposed to confirmed positive cases, quarantining them, following up with them to ensure rapid isolation, and, finally, testing and treating them in case they develop symptoms [9]. This approach effectively controlled COVID-19 as long as quick and efficient processes were followed [10]. A way to guarantee such efficiency was to include digital technologies, particularly mobile phone–based tools, which are widely available worldwide [11]. In the last year, a few systematic literature reviews of COVID-19 apps mentioned contact-tracing apps as an essential type of app used in the context of the pandemic to curb virus transmission [12-17]. On the basis of the experience with the Ebola [18] and H1N1 [19,20] viruses, digital technologies have been increasingly used to support governments in carrying out manual contact-tracing activities. Several conceptual papers and overviews exist on mobile apps for COVID-19 contact tracing [21-27]. There are also a few systematic reviews on the topic [28-30], including a Cochrane review [30] and a literature review [29], focusing on digital contact tracing. The Cochrane review analyzed technologies used in epidemics and was updated in May 2020 to include new COVID-19–related studies. This review showed that such technologies are most effective when used to complement rather than substitute manual contact-tracing activities [30]. A literature review by Jalabneh et al [29] identified 17 apps that could be used for contact tracing and mentioned the use of these apps to help governments contain the pandemic.

### Digital Contact Tracing

According to the Centers for Disease Control and Prevention (CDC), 2 main types of digital contact-tracing tools are used for case management and proximity-tracing or exposure notification apps [31]. Case management tools involve apps and devices that health workers involved in contact-tracing activities can use to capture data and manage contact databases of people tested for the virus. When a person tests positive, contact tracers interview them to recall where, when, and with whom they have been. The contacts are then triaged for assignment to case managers who call and notify contacts, providing options for testing, self-isolation, and referral to a health care provider if necessary. This activity can be done manually and on paper-and-pencil forms, so the technology allows for the streamlining of the process of data entry and management.

Proximity-tracing or exposure notification apps are designed for citizens who voluntarily download and activate such apps to assist in contact-tracing efforts. These apps rely on Bluetooth technology or location-based information stored on the phone to estimate the distance and duration of an encounter between users [12]. The phones exchange alphanumeric strings or keys via Bluetooth that contain such information. This information can be stored on the phone only (decentralized framework) or on a central server (centralized framework) and retained for a limited amount of time [12,32]. Depending on the type of framework, once a positive case is identified, the user or the central server flag their profile as positive, triggering the network and urging them to take action and get tested, self-isolate, or seek the help of health care professionals. This way, exposure notification apps can expand the reach of traditional manual contact tracers, who may fail to identify cases. The apps can reduce the burden on public health staff by allowing for the electronic self-reporting by cases and contacts or by using location data or other features to identify community contacts unknown to the case to look at possible exposure to the virus. This study focuses on proximity-tracing or exposure notification apps as these are designed for citizens.

### Use and Application of Contact-Tracing Apps

Many governments have developed digital contact-tracing apps following international guidelines (eg, the World Health Organization [33], CDC [31], or European CDC [34]) and Google and Apple exposure notification frameworks [35]. For example, as of May 28, 2020, when we started working on this project, we identified 36 apps by searching in the Google Play and Apple App Stores. On the same date, the page entitled *COVID-19 apps* on Wikipedia [36]—which was created on April 1, 2020—included information on 37 contact-tracing apps. As of June 15, 2020, there were already 68 apps and, by December 2, 2020, the page included approximately 100 apps. A recent review of COVID-19 apps in the Google Play and Apple App Stores identified 51 contact-tracing apps available until May 2, 2020 [13]. In the same period, the Technology Review of the Massachusetts Institute of Technology (MIT) launched the *Covid Tracing Tracker* project [37] with the purpose of monitoring and evaluating existing contact-tracing apps. A recent literature review published in July 2020 identified 17 apps in 15 countries [29], whereas Wen et al [38] analyzed 51 apps.

When can contact-tracing apps be considered effective? According to a seminal conceptual paper by Ferretti et al [25], contact-tracing apps can be deemed effective when at least 60% of the population uses them. More than a year after the COVID-19 pandemic was declared, some reviews asked the following question: Are these apps used by individuals [39]? Although many calls for evaluations of contact-tracing apps have been made [40], the evidence about contact-tracing app adoption and effectiveness is scant. A scoping review by Thorneloe et al [41] reported only a couple of examples of apps used by 10% to 20% of the population using data reported in news media outlets. Similar numbers were presented in an overview of contact-tracing apps [16] that provided descriptive information on 14 apps based on publicly available information. The authors focused on technical characteristics (eg, centralized or decentralized frameworks, tracing technology, and technical flaws) and the proportion of the country’s population that used the apps, showing wide ranges (between 0.1% for *BlueZone* Vietnam and 60% for the Chinese *Health Code* used on Alipay and WeChat) [16]. In another conceptual paper, Seto et al [42] argued that the concept of privacy is context-specific and that there is a trade-off between privacy and public health value. To the best of our knowledge, the only comprehensive evaluation of contact-tracing apps is a longitudinal study involving the German *Corona-Warn-App* [43], one of the most downloaded contact-tracing apps in Europe totaling 26.5 million downloads as of March 25, 2021 [44].

How can this low global uptake be explained? The study on the German *Corona-Warn-App* by Munzert et al [43] reported a differential app uptake depending on the users’ self-reported sociodemographic and behavioral profiles. For example, app use was positively associated with older age (≥50 years), education, socioeconomic status, health preconditions, and other preventive behaviors (eg, hand hygiene and mask wearing). App uptake was also higher among those who reported positive cases in their social network or who lived in areas of known outbreaks [43]; it was also higher among users who trusted the national government, the health care system, and science in general, and among those with a strong digital literacy who were less concerned about privacy [43].

### Privacy and Transparency in Data Protection

Privacy, data protection, and the problem of trust in the government appear to be issues of concern, as reported in the aforementioned scoping review [41] and Cochrane review [30]. Numerous conceptual papers in the system design literature have discussed the issue of privacy [45-47], mainly focusing on the use of tracing techniques (eg, location-based vs Bluetooth [48]) and on the use of centralized versus decentralized frameworks, urging some researchers to develop their privacy-preserving apps and frameworks [49]. Decentralized models are privacy-preserving by design; however, they are generally inefficient in responding to the needs of public health systems as they rely on individual users’ willingness to notify the network, which might never occur or might happen with delays that cannot be sustained when dealing with a highly transmissible virus such as SARS-CoV-2 [50]. Conversely, a recent simulation study showed that centralized models could be effective only when 80% of the population uses these technologies [51]. However, centralized models might discourage uptake among users who do not trust the organizations managing the centralized database. In their seminal paper, Ferretti et al [25] argued that app designers and governments supporting contact-tracing apps should be guided by ethical principles (eg, beneficence, reducing misery, equity, and social justice) and follow transparent practices to generate trust in citizens and promote app uptake. Transparency could be achieved, for example, by creating independent oversight advisory boards, publishing the code of the app and the algorithms used, integrating evaluation and research by third parties, and clearly communicating privacy and data protection principles. A way to express such principles is to use the apps’ privacy policy documents, whose availability is requested by the main app stores and recommended by numerous institutions, including the Privacy Trust Framework [52]; the US Federal Trade Commission [53]; and the General Data Protection Regulation (GDPR) of the European Union, which entered into force as of May 2018 [54]. The general recommendation for developers is to produce privacy policy documents that are clear and easy to understand. A way to ensure clarity and comprehensibility of documents is to provide a low readability level, which has been previously considered an element for evaluating apps’ privacy policies [55]; for instance, the Privacy Trust Framework recommends a reading grade level of ≤12 and a Flesch reading ease of 45 [52]. A recent paper investigating contact-tracing apps [56] reported that transparency in the documentation was perceived as an essential element of trust in the apps and developers.

Beyond the conceptual and normative debates among scholars, are citizens’ concerns about privacy real? Are contact-tracing apps truly invasive of privacy? Are the developers or governments behind the apps able to provide transparent and clear information about data protection and treatment? The answers to these questions do not appear in the existing literature on contact-tracing apps. There are a few systematic reviews of COVID-19 apps that mention contact-tracing apps as a type used in the context of the pandemic [12-17]. There are also a number of reviews of COVID-19 contact-tracing apps. For example, the aforementioned MIT *Covid Tracing Tracker* project [37] provides some descriptive information on the technological infrastructure and uptake of these apps. The other 2 overviews of COVID-19 contact-tracing apps [12,16] describe general vulnerabilities instead of considering privacy concerns using the information included in the privacy policy documents. These reviews do not provide a comprehensive, specific analysis of the permissions and data protection [16]. A more recent review of COVID-19 contact-tracing apps [38], published as a conference proceeding, focused on the user privacy aspects, potential data leakage, and other technical features of a sample of 41 apps. The authors mentioned the role of transparency to ensure uptake but did not investigate app characteristics that could enhance transparency beyond publishing the source code, an element present in a few open-source apps analyzed. Another content analysis of contact-tracing apps [57] looked at the public perception of these apps through user reviews and at the number of downloads, tackling the issue of privacy-by-design. Another review analyzed which permissions are needed to allow tracking and tracing and whether the apps have embodied principles of privacy and data protection by design [58]. Another review focused on apps developed in the United States and on usability and qualitative features [59]. Finally, another review looked at the readability of contact-tracing apps [60] without looking at privacy aspects. In conclusion, none of these reviews of contact-tracing apps includes a combined analysis of privacy and data protection principles.

Furthermore, in April 2020, our research group embarked on a project that a few months later resulted in the creation of a nationwide contact-tracing app (Ma3an) [61] in collaboration with the local Ministry of Public Health. Parallel to this project, we searched app databases to identify benchmark apps and used them as a reference for privacy-preserving contact-tracing apps. This was one of the main drivers urging us to undertake a comprehensive systematic review of contact-tracing apps and focus on data protection and privacy aspects.

This study aimed to identify, map, and evaluate all available COVID-19 contact-tracing apps developed worldwide in a systematic way. The specific objectives of this study were to (1) identify and map existing contact-tracing apps; (2) evaluate the type of data collected to define the risks to users’ privacy based on the permissions required; and (3) evaluate the readability and content of privacy policy documents to establish whether these documents transparently communicate details about privacy, data protection, management, and retention. Finally, after more than a year of implementation of the search protocols, data extraction, and assessment, we decided that it was time to respond to the recent call for COVID-19 contact-tracing app evaluations launched by Colizza et al [40] on Nature Medicine, February 15, 2021.

## Methods

We conducted a systematic review of information about existing COVID-19 contact-tracing apps following a rigorous process of app identification, selection, data extraction, and analysis as used in a previously published app review by MB [62] and similar studies targeting different kinds of apps [63,64]. In addition, to address the research objectives, we performed a content analysis of contact-tracing apps’ publicly available Google Play pages and associated privacy policy documents.

### Searches and Sources of Information

We used two main strategies to identify contact-tracing apps: (1) searching for keywords in the Google Play and on the Apple App Store using Google and (2) scanning the list of apps included in 5 websites identified via Google search.

For the first search strategy, we applied the following two search queries: (1) “allintext:COVID-19|covid|covid19|coronavirus AND tracing|exposure site:play.google.com” and (2) “allintext:COVID-19|covid|covid19|coronavirus AND tracing|exposure site:play.google.com.” We conducted the initial searches on May 7, 2020, and updated them almost monthly, on June 1, 2, and 24, 2020; August 18, 2020; November 27, 2020; April 8, 2021; August 7, 2021; and October 31, 2021.

The second search strategy consisted of scanning 5 webpages containing lists of COVID-19 apps, such as the Wikipedia page on COVID-19 apps (first published on April 1, 2020, and last edited on October 20, 2021) [36]; the MIT *Covid Tracing Tracker* project (first published on May 7, 2020, and last updated on January 25, 2021) [37]; the database of contact-tracing apps of the Council of Europe (last updated on June 10, 2020, and then discontinued) [65]; an article on *COVID tracing app roundup* on Android Police (published on September 1, 2020, and last updated on November 21, 2020) [66], with 26 US states using Google Exposure Notification System (ENS), 37 international apps using the same ENS system, and 30 apps not using the ENS framework; and the *List of countries using Google and Apple’s COVID-19 Contact Tracing API* on the XDA Developers website (published on June 24, 2020, and updated on February 25, 2021) [67]. All of these sources were last checked on October 31, 2021.

### Inclusion Criteria

To be included, the apps had to (1) be explicitly aimed at COVID-19 *contact tracing* or *exposure notification*, (2) have a publicly available page on the Google Play or Apple App Stores, and (3) have information on permissions and a privacy policy document available from Google Play. Therefore, we excluded apps designed for contact tracing not explicitly made for COVID-19 that provided general information on COVID-19 or that were *symptom checkers* without mentioning contact-tracing features. We also excluded apps that had an available page only on the Apple App Store as the pages do not include information on permissions as in the Google Play. We also excluded apps if their privacy policy documents were not available (eg, through a broken link) or that did not include a privacy policy explicitly related to the app.

### App Selection Process

We followed a multistage selection process. MB exported the Google search results by looking at the Apple App Store and Google Play in Microsoft Excel. MB then screened the links for relevance, and MAD confirmed the selection. Next, we resolved all disagreements through discussions. Finally, we entered the Google Play links in the Exodus Privacy database [68], which is the auditing platform for Android apps. The Exodus platform looks for embedded trackers (a software meant to collect user data) and permissions requested by each app. An app was excluded if the link to the Exodus database was not working.

### Data Extraction

#### App Characteristics

We extracted the following information from Google Play pages: number of installs, a link to the privacy policy document, 5-star reviews, number of reviews, version of the app, version of the operating system, sponsor, and permission designations. From the Apple App Store page (if available), we extracted the following information: 5-star ratings and number of ratings, app version, seller, operating system version, and language. MB extracted the information, and MAD double-checked it. Any discrepancies were flagged and resolved through discussion.

#### Permission Data

In total, 4 authors (MB, MAD, NM, and RAS), in pairs and independently, extracted the information on permissions using a standardized web-based extraction form based on Exodus reports [68]. All permission items were entered as binary values (1=yes; 0=no). Overall, the raters achieved excellent interrater agreement (percentage of agreement=98.3%; Cohen κ=0.954; Krippendorff α=0.953). All disagreements were resolved through discussion. The Exodus reports [68] label permissions according to 2 levels of risk, as described on the Android developers' page [69]: *Normal* or *Dangerous*, including *Signature* and *SignatureOrSystem*. As described in the book *Android Application Security Essentials* [70], normal permissions are those that “cannot do much harm to the user. They generally do not cost users money, but they might cause users some annoyance...These permissions are automatically granted to the app.” Dangerous permissions are always shown to the user as “they can cause user privacy or financial loss.” Signature permissions allow 2 apps authored by the same developer to access each other’s components. This type of permission is automatically granted to the app if it has the same certificate as the app that declared the permission. Signature or system permissions are “granted to applications with the same certificate as the app that defined the permission. In addition, this protection level includes an app with the same certificate as the Android system image. This permission level is mainly used for applications built by handset manufacturers, carriers, and system apps. These permissions are not allowed for third-party apps. These permissions let apps perform some very powerful functions” [70].

#### Privacy Policy Data Extraction

For apps with available privacy policy information, if the document was in a language other than English, it was translated using Google Translate and saved in PDF format with a timestamp. Similar to the procedure for extracting permission information, 4 authors (MB, NM, MAD, and RAS) independently completed a privacy policy assessment using a standardized web-based checklist. The checklist was adapted from a similar study focusing on data security and privacy in mobile apps addressing depression [71]. The inventory contained a total of 13 specific items (Table 1), which we grouped into 3 main categories: 4 items were in the *privacy* category; 6 items were in the *data management* category; and 3 items were in the *legal framework* category for data protection (eg, the GDPR for European countries or any other framework) explicitly mentioned the right to delete or edit the data, which should be clarified in the legislative framework. We rated each item on a nominal scale (yes=1, no=0, or not applicable, depending on the item).

For this data extraction task, we conducted first a calibration exercise with a sample of 15 randomly selected apps to ensure sufficient reliability and adjust the instrument before applying it to the remaining set of apps. The aforementioned 4 authors individually and independently completed the same checklist. The exercise yielded a sufficient level of agreement (84.8%) as well as reliability indexes (Cohen κ=0.823; Krippendorff α=0.696). Disagreements were resolved through discussion, which allowed for the clarification of a few interpretation issues. After we resolved the disagreements, the 4 raters independently completed the checklist for other apps. The interrater reliability notably improved (percentage of agreement=87.5%; Cohen κ=0.749; Krippendorff α=0.749). Finally, the reviewers completed the data extraction for the remaining apps in pairs. As in the previous task, we resolved disagreements through discussion until we reached a consensus.

**Table 1:** Privacy policy checklist and rubric used to calculate the Privacy Transparency Index (0-100).

Domain and items		Score
**Privacy (25 points)**		
	Does the app collect personally identifiable information?	Yes=0; partial^a^=5; no=10
	Does the privacy policy mention that the app can be used without entering identifiable information?	Yes^b^=5; no=0
	Does the privacy policy mention that the app collects identifiable information such as full name, email, and phone number?	Yes or (N/A^c^)^b^=5; no=0
	Does the privacy policy mention that the app provides the option of a personal identification number, password, or log-in process to view and enter user data?	Yes or N/A^b^=5; no=0
**Data management (50 points)**		
	Does the privacy policy explicitly state which type of data are processed?	Yes=15; no=0
	Does the privacy policy contain a section on “how the app works” explicitly?	Yes=5; no=0
	Does the privacy policy state that the app or server encrypts the entered data?	Yes=10; no=0
	Does the privacy policy describe the process of data exchange and communication between server and phone related to user-entered information?	Yes=5; no=0
	Does the privacy policy state that the user information is stored on the phone or device?	Yes=10; no=0
	Does the privacy policy mention data retention?	Yes=5; no=0
**Legal framework (25 points)**		
	Does the privacy policy mention the GDPR^d^? If not, does the privacy policy mention other legislative frameworks?	Yes=15; no=0
	Does the privacy policy state whether users can delete entered information?	Yes=5; no=0
	Does the privacy policy state whether users can edit entered information?	Yes=5; no=0

^a^In this context, partial information is related to the use of location services only.

^b^Not applicable options for apps that do not collect personal or identifying information.

^c^N/A: not applicable.

^d^GDPR: General Data Protection Regulation.

### Data Elaboration

#### App Characteristics

On the basis of the information reported on the Google Play page or on the other sources we used, we categorized the apps by country and continent according to the NationsOnline classification [72]. We also categorized the apps by the type of coverage (country or state, county, or city, depending on their geographical coverage) and type of sponsor (government; nonprofit organization; profit organization; and multistakeholder, involving a combination of the previous categories). Finally, we grouped the apps with the associated Google Play information according to the number of installs (ranging from ≥50 to ≥100 million) as a relative measure of popularity.

#### Permission Data

We counted the number of trackers and permissions identified through the Exodus platform [68]. Next, we assigned numeric values to each protection level: normal permissions=1; dangerous, signature, or system permissions=2; and trackers=3 as they constitute a higher level of danger to users’ privacy. We then multiplied the number of permissions and trackers by the protection level to calculate a *Permission Accumulated Risk Score*. The higher this score, the higher the risk.

#### Privacy Policy Data

We assigned different points to the aforementioned checklist (Table 1) to calculate a *Privacy Transparency Index*, which could range from 0 to 100. Similar to the Permission Accumulated Risk Score, the higher the Privacy Transparency Index, the more transparent the privacy policy.

We also assessed the readability needed to understand the policy using a combined estimate of readability indexes provided by the Automatic Readability Checker, a web-based readability calculator [73]. This tool outputs an estimate based on 7 popular readability formulas: the Flesch Reading Ease Formula, Flesch-Kincaid Grade Level, Gunning fog index, Simple Measure of Gobbledygook Index, Coleman-Liau Index, Automated Readability Index, and Linsear Write Formula. Naturally, the lower the grade, the easier it is to understand. For example, the Privacy Trust Framework recommends a reading grade level of ≤12 for policy documents [52].

#### Analyses

We used descriptive statistics to summarize the apps’ characteristics. For apps with permission and privacy policy data, we summarized continuous variables (eg, number of ratings, Permission Accumulated Risk Score, and Privacy Transparency Index) using mean and SD or median and IQR for count variables where appropriate. In addition, we investigated potential associations between app characteristics (such as type of sponsors, number of installs, and number of reviews or ratings), Permission Accumulated Risk Score, and Privacy Transparency Index using ANOVAs, chi-square tests, and Pearson correlation tests (significance level was assumed at *P*<.05).

## Results

### Search Results

**Figure 1:**
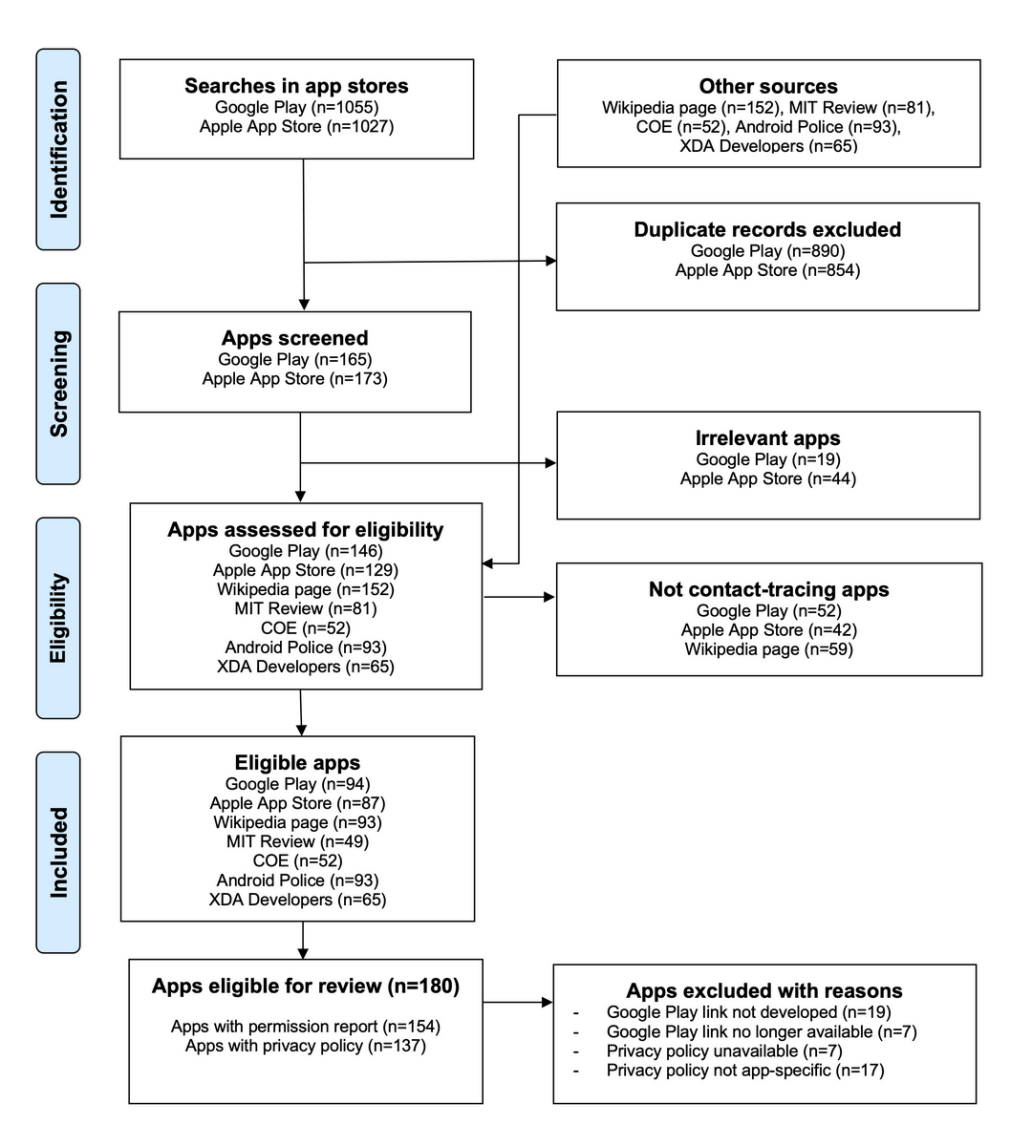
App selection process. COE: Council of Europe; MIT: Massachusetts Institute of Technology.

The selection process is illustrated in Figure 1. We applied the search queries to Google on May 7, 2020; June 1, 2, and 24, 2020; August 18, 2020; November 27, 2020; April 8, 2021; August 7, 2021; and October 31, 2021. We exported 1055 records from Google Play and 1027 records from the Apple App Store in Microsoft Excel. After removing duplicate links, we screened 15.64% (165/1055) of unique app links available from Google Play and 16.85% (173/1027) of links available from the Apple App Store. In this first screening stage, we excluded 11.5% (19/165) and 25.4% (44/173) of apps from Google Play and the Apple App Store, respectively, that were deemed irrelevant as they were not related to COVID-19. The remaining 88.5% (146/165) and 74.6% (129/173) of apps from Google Play and the Apple App Store, respectively, were assessed for eligibility together with 152 apps from the Wikipedia page [36], 81 from the MIT *Covid Tracing Tracker* project [37], 52 from the Council of Europe database of contact-tracing apps [65], 93 from the Android Police page [66], and 65 from the XDA Developers page [67]. Finally, we excluded apps that were not designed for contact tracing (52/146, 35.6% from Google Play; 42/129, 32.6% from the App Store; and 59/152, 38.8% from Wikipedia).

The final list included 180 unique COVID-19 contact-tracing apps that were potentially eligible for review. Of these, 85.6% (154/180) had a Google Play link to generate an Exodus platform permission report [68], and 76.1% (137/180) had an associated privacy policy document. A total of 14.4% (26/180) of the apps did not have a permission report either because a Google Play link was not available (19/26, 73%) or because it was no longer available at the time of the analysis (7/26, 27%). The other 4.5% (7/154) of apps did not have a privacy policy document available, and 11% (17/154) had privacy policy documents that were not app-specific. Of the 180 selected apps, 132 (73.3%) contained data related to both permissions and privacy policies. A complete list of all 180 identified apps up to October 31, 2021, is included in Multimedia Appendix 1. The list contains links to the Google Play pages and to the Exodus platform reports. The list is also publicly available on Tableau Public from the link [74].

**Figure 2:**
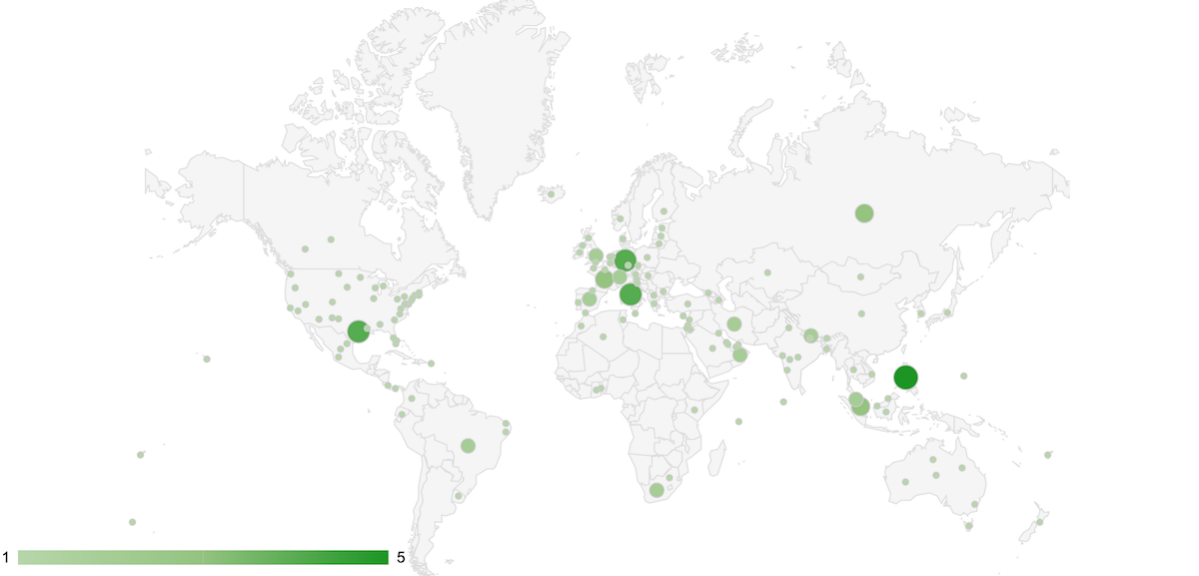
Geographic distribution of the identified contact-tracing apps, aggregated by country.

The identified 180 apps covered 152 geographical units (countries or regions, states or provinces, counties, territories, or cities) in 90 different countries spanning all 5 continents. Most apps came from the Americas (53/180, 29.4%), Asia (53/180, 29.4%), and Europe (46/180, 25.6%). The African continent had 6.1% (11/180) of the apps, Oceania had 5% (9/180), and 4.4% (8/180) of the apps covered multiple continents or were developed to cover different countries. The world map in Figure 2 represents the global distribution of the COVID-19 contact-tracing apps. The larger the bubble, the higher the number of apps for each country.

The United States had the highest absolute number of apps as 20% (36/180) were developed to cover different states. This number does not include 1.1% (2/180) of the apps, which came from the US unincorporated territories of Guam (*Guam Covid Alert*) and Puerto Rico (*Rastrea el Virus*). A total of 1.1% (2/180) of the apps (*Care19 Alert* and *Care19 Diary*) covered the states of North Dakota, South Dakota, and Wyoming. The country with the second-highest number of apps was Australia (6/180, 3.3%). Germany, Great Britain, India, and the Philippines had 2.8% (5/180) of the apps each; Brazil and Italy had 2.2% (4/180) of the apps each; France, Malaysia, Mexico, Nepal, Russia, Singapore, South Africa, and Spain had 1.7% (3/180) of the apps each; and Canada, Iran, the Netherlands, Oman, Switzerland, and the United Arab Emirates had 1.1% (2/180) of the apps each.

Most contact-tracing apps were sponsored by governments (132/180, 73.3%), followed by private organizations (28/180, 15.6%) and nonprofit organizations (14/180, 7.8%). A small number of apps involved multiple stakeholders, including consortia of private, nonprofit, and governmental organizations (6/180, 3.3%).

### App Characteristics

The list of 154 apps with permission data is provided in Multimedia Appendix 2 (tab 1). In Table 2, we report the basic descriptive information for the sample of apps grouped according to the number of installs. As of October 8, 2021, based on Google Play install categories, the 154 apps totaled 264.5 million installs (1.7 million on average), ranging from 10 to 100 million. The most installed app was *AarogyaSetu*, developed by the Indian National Informatics Centre eGov Mobile Apps department. The least installed app was *Aggie-COVID-19*, which was designed for New Mexico University. Most apps were installed between 100,000 and 1 million times (106/154, 68.8%), with approximately one-third being installed <100,000 times (48/154, 31.2%).

Most apps were developed by governments (116/154, 75.3%), private organizations (17/154, 11%), nonprofit organizations (11/154, 7.1%), and multistakeholder consortia (10/154, 6.5%). No significant association between the type of sponsor and number of install categories was detected. The average rating was 3.5 (SD 0.7) on Google Play and 3.6 (SD 0.9) on the Apple App Store based on a subsample of 120 apps with a valid App Store page. The average number of reviews was 26,412 (SD 143,803) on Google Play and 5120 (SD 28,826) on the App Store.

The average number of reviews on Google Play was significantly associated with the number of installs (*F*_3_=5.04; *P*<.001; η^2^=0.07), with apps installed ≥1 million times receiving more reviews than those installed between 100,000 and 500,000 times and more reviews than those installed <100,000 times. We detected a similar difference in the number of ratings on the Apple App Store (*F*_3_=3.59; *P*=.02; η^2^=0.05); in this case, the average number of ratings was significantly higher in apps installed <100,000 times and those installed between 500,000 and 1 million times.

**Table 2:** Characteristics of the sample of apps organized by the number of installs (N=154).

Variable				Total		Number of installs								*P* value^a^	
						≥1 million (n=49)		500,000 to 1 million (n=12)		100,000 to 500,000 (n=45)		<100,000 (n=48)			
**Type of sponsor, n (%)**															.05
	Government		116 (75.3)		42 (85.7)		10 (83.3)		36 (80)		28 (58.3)				
	Private		17 (11)		2 (4.1)		1 (8.3)		3 (6.7)		11 (22.9)				
	Nonprofit		11 (7.1)		4 (8.2)		1 (8.3)		3 (6.7)		3 (6.3)				
	Multistakeholder		10 (6.5)		1 (2)		0 (0)		3 (6.7)		6 (12.5)				
**Average app ratings, mean (SD; range)**															
	Google Play		3.49 (0.69; 1.00-5.00)		3.53 (0.72; 1.00-4.70)		3.16 (0.65; 1.80-4.10)		3.41 (0.62; 1.30-4.40)		3.61 (0.71; 1.70-5.00)		.17 (.13)		
	Apple App Store^b^		3.59 (0.94; 1.00-5.00)		3.46 (0.97; 1.00-4.90)		3.42 (1.15; 1.20-5.00)		3.69 (0.91; 1.60-5.00)		3.74 (0.86; 1.50-5.00)		.52 (.64)		
**Average number of ratings, median (IQR; range)**															
	Google Play		972 (5094; 1-1,600,000)		16,373 (32,382; 960-1,600,000)		2362 (1200; 1553-4094)		750 (569; 155-3275)		114 (137; 1-1135)		<.001 (<.001)		
	Apple App Store^b^		103 (591; 1-287,200)		1047 (3553; 1-287,200)		464 (763; 4-1400)		112 (226; 1-2200)		25 (47; 1-595)		.02 (<.001)		
**Permission data, median (IQR; range)**															
	Average number of permissions		9 (10; 2-44)		10 (9; 6-40)		7 (6; 6-44)		8 (7; 2-42)		12 (12; 4-41)		.27 (.19)		
	Average percentage of dangerous permissions		13 (21; 0-63)		13 (21; 0-36)		0 (20; 0-63)		11 (19; 0-50)		15 (21; 0-44)		.87 (.50)		
	Average number of trackers		1 (2; 0-11)		2 (2; 0-7)		0 (1.3; 0-4)		0 (2; 0-5)		1 (1.25; 0-11)		.38 (.23)		
	Permission Accumulated Risk Score		16 (26; 4-74)		14 (26; 6-63)		10 (13; 6-74)		14 (22; 4-70)		23.5 (26; 4-65)		.34 (.11)		
**Privacy policy data, n (%)**															.76
	Privacy policy available		132 (85.7)		44 (89.8)		10 (83.3)		37 (82.2)		41 (85.4)				
	Privacy policy unavailable		22 (14.3)		5 (10.2)		2 (16.7)		8 (17.8)		7 (14.6)				
**Readability^b^**															
	Grade level, median (IQR; range)		12 (3; 7-23)		12 (4; 8-23)		11 (2; 7-16)		12 (3; 7-19)		12 (2; 8-18)		.07 (.13)		
	**Readability level, n (%)^b^**													.50	
		Very difficult to read	14 (9.1)		7 (15.9)		1 (10)		4 (10.8)		2 (4.9)				
		Difficult to read	67 (43.5)		24 (54.6)		4 (40)		18 (48.7)		21 (51.2)				
		Fairly difficult to read	47 (30.5)		11 (25)		4 (40)		14 (37.8)		18 (43.9)				
		Standard or average	4 (2.6)		2 (4.6)		1 (10)		1 (2.7)		0 (0)				
Policy—transparency index, median (IQR; range)^b^				55 (30; 5-95)		60 (31.3; 25-95)		60 (36.3; 20-90)		60 (35; 5-85)		50 (20; 5-90)		.65 (.68)	

^a^*P* value for independent sample *t* tests (2-tailed), chi-square tests, or *F* tests comparing the number of install categories and the other variables. The *P* value for the Kruskal-Wallis test, the nonparametric equivalent of an ANOVA, is indicated in parentheses.

^b^The calculations are available from a total of 132 apps with privacy policy documents.

### Permission Data

The typology of permissions, identified through the Exodus platform automatic permission extraction, is presented in Multimedia Appendix 3 (tab 2). Across the 154 apps with valid permission data, there were 94 different types of permissions, of which 17 (18%) were dangerous or special.

Among the normal permissions, the one used in all apps was *Internet, have full network access* (154/154, 100%). The permissions used by more than half of the apps were *view network connections* (150/154, 97.4%); *wake lock, prevent phone from sleeping* (142/154, 92.2%); *run in foreground* (137/154, 89%); *run at startup* (131/154, 85.1%); and the permissions related to Bluetooth as *pair with Bluetooth devices* (118/154, 76.6%). The most frequently used dangerous permission was *access precise location (GPS and network-based)*, which was used by approximately half of the apps (73/154, 47.4%). Other dangerous permissions used by approximately one-third of the sample included *access approximate location (network-based)* (57/154, 37%), *take pictures and videos* (51/154, 33.1%), and *modify or delete the contents of your SD card* (44/154, 28.6%). On average, each app collected 9 permissions (IQR 10, range 2-44). Only 0.6% (1/154) of the apps collected 2 permissions (*TRACE Taguig,* the Philippines), and only 0.6% (1/154) collected 44 permissions (*Shlonik*, Kuwait); 46.1% (71/154) of the apps required fewer permissions.

The average proportion of dangerous permissions was 13% (IQR 21%, range 0%-63%). A total of 39% (60/154) of the apps did not use any dangerous permissions, and 0.6% (1/154) reported using the most dangerous permissions (*Corona Watch*, Karnataka province, India).

In addition, the Exodus platform extracted approximately 30 different trackers (Multimedia Appendix 3, tab 3). Google Firebase Analytics was the most frequently used tracker (80/154, 51.9%), followed by Google CrashLytics for crash reporting (48/154, 31.2%). Although some apps had analytics and app statistic information trackers, others had trackers used to profile users (eg, Facebook log-in, Segment, AltBeacon, and DOV-E) or for advertising (Google AdMob; 6/154, 3.9%). On average, each app used 1 tracker (IQR 2, range 0-11). Although 41.6% (64/154) of the apps did not use any trackers, 0.6% (1/154) used the most trackers (*Citizen SafePass*).

On the basis of the number and type of permissions and trackers, the average Permission Accumulated Risk Score was 16 (IQR 26, range 4-74). Of the 154 apps, 2 (1.3%) scored the lowest—*TRACE Taguig* (the Philippines) and *Beat COVID Gibraltar*—and 1 (0.6%) scored the highest—*Shlonik* (Kuwait). Approximately one-fifth of the sample (40/154, 26%) obtained the second- and third-lowest Permission Accumulated Risk Score (score of 6: 23/154, 14.9%; score of 7: 17/154, 11%).

### Privacy Policy Data

Privacy policy data extraction was available for 85.7% (132/154) of the apps, as 14.3% (22/154) did not have a working privacy policy link or document. A spreadsheet containing the privacy policy data extraction for each app is available in Multimedia Appendix 4.

Regarding readability, the privacy documents required a median grade level of 12 (IQR 3, range 7-23). We found the lowest level in the privacy policy documents of *Stopp Corona* (Austria) and *The Territory Check-In* (Northern Territory, Australia) and the highest level in the policy document of the *Taiwan Social Distancing* app.

Most of the privacy policy documents were *difficult* or *very difficult to read* (81/132, 61.4%), with approximately one-third being *fairly difficult to read* (47/132, 35.6%). Only 3% (4/132) of the apps had a *standard or average* reading level. In addition to *Stopp Corona* and *The Territory Check-In,* the other 2 apps were *COVID Alert* (South Africa) and *COVID Alert* (Canada).

The sample distribution according to the privacy policy checklist items is shown in Table 3. Notable strengths in terms of privacy included the fact that most policy documents explicitly stated when personal identifiers were collected (116/132, 87.9%) and what type of data was collected and for how long (100/132, 75.8%). In addition, privacy policy documents mentioned that these data were protected through a personal identification number or password (78/132, 59.1%). Nevertheless, most apps collected or partially collected personally identifiable information (89/132, 67.4%). Other limitations of data management included the fact that most privacy policies did not have a section clearly explaining how the app worked (86/132, 65.2%), did not state or explain how the app or server encrypted the data, or did not describe the process of data exchange (105/132, 79.5%).

In terms of the legal framework used, most policy documents mentioned that they abided by the GDPR or other national-level legislative data protection frameworks (82/132, 62.1%). Another notable strength of the right to be forgotten is that most of the policy documents stated that the users had the right to delete the app or their profile (90/132, 68.2%). Nevertheless, a few policies mentioned the right to rectify or edit the profile (50/132, 37.9%).

On the basis of the privacy policy checklist, the average Privacy Transparency Index was 56 (SD 22, range 5-95), which can be considered moderate as it is slightly above the median value of 50. Of the 132 apps, 4 (3%) scored the lowest Privacy Transparency Index—*The Territory Check-In* (Australia); *Bardghat Municipality - COVID-19/Disaster Response* and *Bharatpur Metropolitan|COVID-19 Response System* (both from Nepal); and *Check On the other hand, oneTAS* (Australia)—1 (0.8%) scored the highest Privacy Transparency Index—*COVID Tracker Ireland*—and 5 (3.8%) scored the second-highest Privacy Transparency Index (90/100)—*Corona-Warn-App* (Germany), *NHS COVID-19 App* (the United Kingdom; 2 versions, one pilot and one national), *SwissCovid* (Switzerland), and *Protect Scotland*.

**Table 3:** Completed checklist of the Privacy Transparency Index applied to 132 apps.

Domain, item, and score				Apps, n (%)
**Privacy**				
	**Does the app collect personally identifiable information?**			
		Yes=0	79 (59.8)	
		Partial^a^=5	10 (7.6)	
		No=10	43 (32.6)	
	**Does the privacy policy mention that the app can be used without entering identifiable information?**			
		Yes or N/A^b^=5	48 (36.4)	
		No=0	84 (63.6)	
	**Does the privacy policy mention that the app collects identifiable information such as full name, email, and phone number?**			
		Yes or N/A=5	116 (87.9)	
		No=0	16 (12.1)	
	**Does the privacy policy mention that the app provides the option of a personal identification number, password, or log-in process to view and enter user data?**			
		Yes or N/A=5	78 (59.1)	
		No=0	54 (40.9)	
**Data management**				
	**Does the privacy policy explicitly state which type of data are processed?**			
		Yes=15	100 (75.8)	
		No=0	32 (24.2)	
	**Does the privacy policy contain a section on “how the app works” explicitly?**			
		Yes=5	46 (34.8)	
		No=0	86 (65.2)	
	**Does the privacy policy state that the app or server encrypts the entered data?**			
		Yes=10	57 (43.2)	
		No=0	75 (56.8)	
	**Does the privacy policy describe the process of data exchange and communication between server and phone related to user-entered information?**			
		Yes=5	27 (20.5)	
		No=0	105 (79.5)	
	**Does the privacy policy state that the user information is stored on the phone or device?**			
		Yes=10	51 (38.6)	
		No=0	81 (61.4)	
	**Does the privacy policy mention data retention?**			
		Yes=5	100 (75.8)	
		No=0	32 (24.2)	
**Legal framework**				
	**Does the privacy policy mention the GDPR^c^? If not, does the privacy policy mention other legislative frameworks?**			
		Yes=15	82 (62.1)	
		No=0	50 (37.9)	
	**Does the privacy policy state whether users can delete entered information?**			
		Yes=5	90 (68.2)	
		No=0	42 (31.8)	
	**Does the privacy policy state whether users can edit entered information?**			
		Yes=5	50 (37.9)	
		No=0	82 (62.1)	

^a^Partial score when the app used location services only.

^b^N/A: not applicable.

^c^GDPR: General Data Protection Regulation.

### Correlations

The correlations among continuous variables representing app characteristics, Permission Accumulated Risk Score, readability, and Privacy Transparency Index are shown in Table 4. There was a small significant correlation between the average app ratings in the 2 app stores (*r*=0.21; *P*=.02; 116/154, 75.3%). Similarly, there was a larger, highly significant correlation between the number of ratings reported in the Google Play and Apple App Stores (*r*=0.87; *P*<.001; 116/154, 75.3%), which, in turn, was significantly correlated with the number of installs (Google Play ratings: *r*=0.96; *P*<.001; 150/154, 97.4%; Apple App Store ratings: *r*=0.90; *P*<.001; 120/154, 77.9%). This finding is consistent with the ANOVA reported at the end of the *App Characteristics* section. The Permission Accumulated Risk Score had a small negative correlation with the average rating on the Apple App Store (*r*=−0.20; *P*=.03; 120/154, 77.9%), suggesting that, the lower the rating, the higher the risk to the users’ privacy. The Privacy Transparency Index was negatively associated with the Permission Accumulated Risk Score (*r*=−0.25; *P*<.001; 132/154, 85.7%), suggesting that, the higher the risk to one’s data, the lower the transparency index of the related policy document.

**Figure 3:**
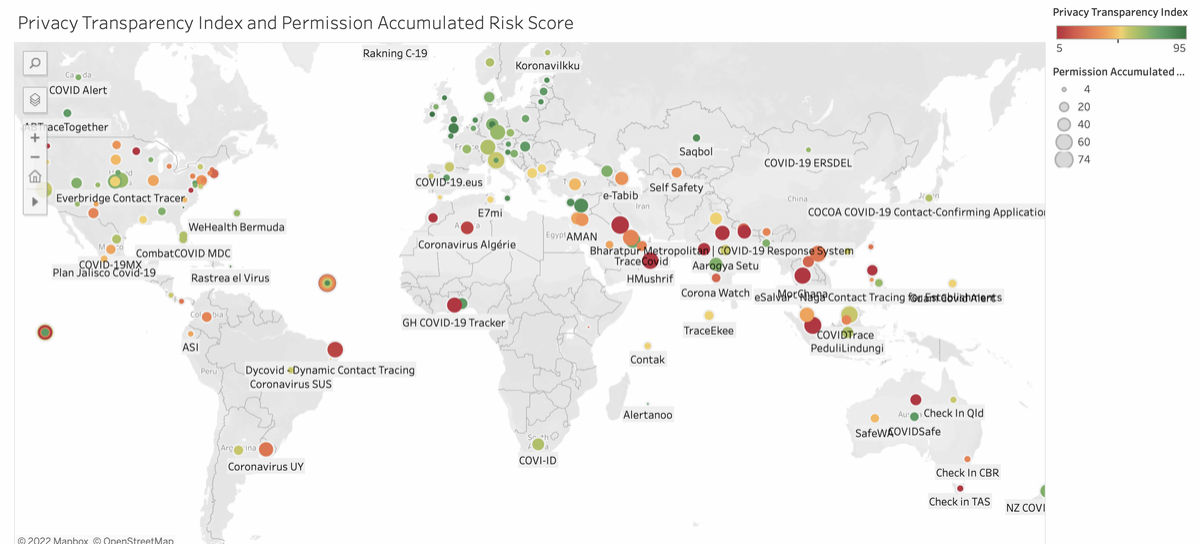
Map representing the Permission Accumulated Risk Score (size of the bubble) and the Privacy Transparency Index (color).

Figure 3 is a screenshot of a map representing the relationship between the Permission Accumulated Risk Score and Privacy Transparency Index. The map is publicly available on Tableau Public [74].

**Table 4:** Correlation table for continuous variables.

Variables	1	2	3	4	5	6	7
1. Average rating (Google Play)	—^a^	—	—	—	—	—	—
2. Average rating (Apple App Store)	0.21^b^	—	—	—	—	—	—
3. Number of ratings (Google Play)	0.04	0.03	—	—	—	—	—
4. Number of ratings (Apple App Store)	0.11	0.13	0.87^c^	—	—	—	—
5. Number of installs	0.02	0.04	0.96^c^	0.90^c^	—	—	—
6. PARS^d^	0.13	−0.20^b^	0.05	0.04	0.01	—	—
7. Grade level (readability)	0.04	0.10	0.05	0.11	0.08	0.02	—
8. PTI^e^	−0.03	0.10	0.00	<0.00	0.02	−0.25^f^	−0.15

^a^Not applicable.

^b^*P*<.05.

^c^*P*<.001.

^d^PARS: Permission Accumulated Risk Score.

^e^PTI: Privacy Transparency Index.

^f^*P*<.01.

## Discussion

### Principal Findings

This paper presents the first systematic review of COVID-19 contact-tracing apps developed worldwide that explored the apps’ approach to data protection and privacy. In addition, we assessed the number and type of permissions requested by the apps and how transparent the privacy policy documents were about data protection rules. This systematic review aimed to (1) identify and map existing contact-tracing apps, (2) evaluate the potential risks to users’ privacy through the assessment of the type and quantity of permissions requested, and (3) evaluate the readability and level of transparency of related privacy policy documents.

We adopted a systematic search, selection, and identification process using different sources [62]. This systematic approach allowed us to identify 180 COVID-19 contact-tracing apps covering 90 countries. Of these 180 apps, 154 (85.6%) had valid links to extract permission data, and 132 (73.3%) had privacy policy documents that could be analyzed. Our search strategy allowed us to generate a much larger sample than those reported in recent COVID-19–related app audits [13,29,38]. Furthermore, the selection of apps we analyzed is more extensive than the one included in the MIT *Covid Tracing Tracker* project [37], which currently comprises 81 apps. The most updated source of information to date is Wikipedia’s *COVID-19 apps* page [36], which lists 152 apps. Although the number of apps for COVID-19 contact tracing might grow over time with more governments embarking on digital contact-tracing efforts, some researchers believe that the momentum is now over considering how the pandemic has evolved. In the absence of zero–COVID-19 strategies, mitigation strategies and vaccination campaigns might take priority over contact tracing and other NPIs [75]. Nevertheless, we hope that this review will spark the interest of the public health and global health community, who might want to contribute to the enlargement and maintenance of the app database, which is already accessible on Tableau Public [74].

### Permission Data and Privacy Risk

To achieve the second objective, we analyzed publicly available information from Google Play webpages and extracted it using the Exodus platform scanner [68]. This objective assessment and data extraction allowed us to systematically identify and classify the types of permissions and their relative risk to users’ privacy. We developed a Permission Accumulated Risk Score to qualify the level of risk, accounting for some dangerous permissions and the presence of invasive trackers. The wide variability in the number and type of permissions and trackers identified across the sample of 154 apps included in this study suggests that there is no single approach to privacy-preserving app development. Consistent with the conclusions of Azad et al [58], many apps seem to collect more permissions than needed, some of which have the potential to breach users’ privacy. Although the number and type of permissions varied across the apps, it seems that some governments are particularly interested in collecting more data than others. On the one hand, most of the apps requested nondangerous permissions such as allowing for full network access, preventing the devices from sleeping, and asking to pair Bluetooth devices. The use of Bluetooth technology for contact tracing seems to be almost ubiquitous [58,76] and has been deemed a privacy-preserving approach [45,48,51]; nevertheless, some apps included very invasive permissions or required constant internet connectivity, which might not be available at all times, making real-time exposure notification difficult or impractical [77].

Moreover, some apps require read-and-write privileges to access the phone storage and camera to use QR codes, an approach that seems appropriate for some types of offline self-check actions for digital contact tracing [77]. Other apps require access to the microphone, GPS location, and phone identity to allow for government operations of contact tracing and network exposure notification. Although it can be efficient from a public health perspective, this approach might generate some general privacy concerns. Our findings show a negative correlation between the Permission Accumulated Risk Score and the average rating of the selected apps on the Apple App Store, which might indicate that users did not like the design or usability or did not trust these apps, expressing a lower rating [78].

### Readability and Transparency of Privacy Policies

Most apps (81/132, 61.4%) included privacy policies that were very difficult to read, suggesting that only educated users could interpret the information presented. This finding is consistent with some studies evaluating the readability of contact-tracing app privacy policies [60] and with other apps for other health domains such as mental health [71,79], health and fitness [63], and general health for young generations [54,55].

When it comes to transparency, of the 180 contact-tracing apps identified, 24 (13.3%) did not include a valid link to a privacy policy document or included a link to a policy document that was not specific to the app. Although not many users might read a privacy policy before or after installing an app, not having such a document available can raise concerns about the developers’ transparency, negligence, or incompetence [79]. Another notable finding was the inverse relationship between the Permission Accumulated Risk Score and Privacy Transparency Index, suggesting that, the higher the risk of violating one’s privacy through app permissions, the lower the level of transparency of the policy document. Although this relationship is based on our *expert assessment* of the documents and the permission data, the data make sense. The data suggest that some developers might collect more data than necessary without feeling the need to communicate this to the users [80].

As trust in governments seems to be dwindling worldwide, it would be expected that contact-tracing apps would follow a truly decentralized framework and be based on transparency and openness principles [25]. Of the 132 privacy policy documents analyzed, most (81/132, 61.4%) achieved an above-average rating in the bespoke Privacy Transparency Index. Most policy documents indicated that the apps collected personal identifiers. Although it provides helpful information about data management, this suggests that a genuinely privacy-preserving and completely anonymous approach to contact tracing may be unfeasible in real-life scenarios [49]. Nevertheless, the privacy-preserving apps (ie, those with low Permission Accumulated Risk Score) had higher ratings on the Apple App Store. Their privacy policy documents had a higher Privacy Transparency Index, suggesting that transparency and privacy can go well together with positive app reviews, which may indicate better user engagement and sustained use.

### Strengths and Limitations

This is the first systematic review and evaluation of COVID-19 contact-tracing apps that combines an assessment of the privacy risk and the privacy policies’ transparency and readability. An essential strength of this study is the methodological approach following a specific protocol for selection, data extraction, and analysis. Another strength is the availability of the data collected across 154 apps developed worldwide. The limitations of this study include the use of bespoke measures to quantify the level of risk (the Permission Accumulated Risk Score) and the level of transparency (the Privacy Transparency Index). Although these instruments require formal validation, we tried to minimize the potential subjectivity and errors by completing a series of trainings and assessing interrater agreement and reliability indexes to establish a good level of agreement in evaluating the apps. Another limitation was the use of data generated from Google Play as some apps were developed only for iOS and were not included in the study. Unfortunately, the App Store for iOS does not include information about the permissions that the apps require; this is due to the different software architecture between iOS and Android. Another limitation is related to the extreme volatility of the mobile app market and its characteristics. We provided a global snapshot of all available contact-tracing apps as of October 31, 2021, after having monitored the market for approximately a year. Considering that the pandemic is still ongoing, existing contact-tracing apps might disappear, new ones could be developed, or different technological solutions could be adopted to provide exposure notifications (eg, merging databases or aligning data exchange protocols between European or US states). This would imply that the existing apps might have different software permissions and privacy policies. Our database provides a historical classification of contact-tracing apps that were developed over more than a year, and we made such a list of apps available from the Tableau link.

### Conclusions

COVID-19 contact-tracing app developers should find a balance between following privacy-preserving frameworks and collecting personal information to serve the needs of public health institutions to ensure efficient and practical support for manual contact-tracing efforts. Developers should reduce the amount of data collected and relate it to the sole purpose of contact tracing. They should also put more effort into making privacy policy documents more accessible and easier to read and providing the information needed to foster trust in governments and institutions for the fight against COVID-19. Better and more useful digital contact-tracing apps would help governments undertake contact-tracing efforts more efficiently and effectively.

## Appendix

Multimedia Appendix 1 List of contact-tracing apps identified (N=180).

Multimedia Appendix 2 Characteristics of selected contact-tracing apps with permission data available (n=154).

Multimedia Appendix 3 Permission data for selected contact-tracing apps (n=154).

Multimedia Appendix 4 Privacy policy assessment of selected contact-tracing apps with an existing valid policy document (n=132).

## Abbreviations

CDC: Centers for Disease Control and Prevention
ENS: Exposure Notification System
GDPR: General Data Protection Regulation
MIT: Massachusetts Institute of Technology
NPI: nonpharmacological intervention
